# Hepcidin promotes osteogenic differentiation through the bone morphogenetic protein 2/small mothers against decapentaplegic and mitogen-activated protein kinase/P38 signaling pathways in mesenchymal stem cells

**DOI:** 10.3892/mmr.2014.2769

**Published:** 2014-10-24

**Authors:** HUADING LU, LIYI LIAN, DEHAI SHI, HUIQING ZHAO, YUHU DAI

**Affiliations:** Department of Orthopedics, Third Affiliated Hospital of Sun Yat-sen University, Guangzhou, Guangdong 510630, P.R. China

**Keywords:** hepcidin, osteogenic differentiation, bone morphogenetic protein/small mothers against decapentaplegic, mitogen-activated protein kinase/P38

## Abstract

The ability of mesenchymal stem cells (MSCs) to differentiate into osteogenic lineages requires management for their future use in treating bone destruction and osteoporosis. Hepcidin is closely associated with bone metabolism, however, it remains to be elucidated whether hepcidin affects osteogenic differentiation in MSCs. The present study demonstrated that hepcidin enhanced osteoblastic differentiation and mineralization, which was manifested by an upregulation in the differentiation markers alkaline phosphatase and osteogenic genes. Furthermore, the expression levels of bone morphogenetic proteins and small mothers against decapentaplegic homologs were concomitantly increased following hepcidin treatment. In addition, the p38 mitogen-activated protein kinase may be an upstream kinase for osteoblastic differentiation. Thus, hepcidin may be important in the osteogenic differentiation of MSCs and may be considered as a target in the development of therapies for pathological bone loss.

## Introduction

Osteoporosis, which is distinguished by a reduction in bone mass and deterioration in bone microarchitecture, is a systemic disease of the skeletal system with increased bone fragility and an increased risk of fracture (1). In total, ~40% of postmenopausal women are affected by osteoporosis and, as the population is ageing, a continual increase in this percentage and the medical and socioeconomic impact is expected (2). The primary cause of postmenopausal osteoporosis is a deficiency in endogenous estrogen (3). However, the widespread use of hormone replacement usually has severe side effects, including sleep disturbances, depressive mood and headaches. In addition, there is an increase in the incidence of endometrial hyperplasia, breast and ovarian cancer (4–7). Alternative drugs with similar therapeutic effects, but with fewer side effects, are being investigated in order to replace estrogen medications. Thus, an improved understanding of the osteogenic processes may provide a basis for therapeutic improvement and to assist in the development of novel therapies against menopausal bone loss.

Osteoporosis is considered to be a disease associated with abnormal calcium metabolism. However, a number of clinical observations have suggested that it is closely associated with the degree of iron overload observed in menopausal women (8,9). Iron overload may repress the formation of osteoblasts in bone and may also stimulate the resorption of bone by osteoclasts (10,11). A previous study demonstrated that iron inhibits the synthesis of gonadotrophs from the anterior pituitary, which resulted in the depression of gonadal hormone formation (12). Systemic iron homeostasis is fundamentally governed by the hepcidin-ferroportin regulatory axis, where the key regulator hepcidin regulates iron homeostasis in chordates (13).

Advances in the understanding of hepcidin, an iron-regulatory hormone, have revealed its importance in the development of inflammation, hereditary hemochromatosis, iron-loading anemia, cancer and chronic kidney disease (14–17). In addition, hepcidin may have effects against osteoporosis by preventing iron overload, which may be associated with increases in intracellular calcium (18). However, the mechanism underlying the effects of hepcidin on bone tissue differentiation and growth remains to be fully elucidated.

Thus, the aim of the present study was to investigate the effects of hepcidin on the osteogenic differentiation of MSCs. An improved understanding of the effects of hepcidin in MSCs during cell osteogenic differentiation may have implications in women’s health following the menopause, potentially contributing to the development of new therapeutic targets for osteoporosis.

## Materials and methods

### Reagents

α-minimal essential medium (α-MEM), fetal bovine serum (FBS), penicillin and streptomycin were obtained from Invitrogen Life Technologies (Carlsbad, CA, USA). Bradford Protein Assay Kit and ethidium bromide solution were purchased from Bio-Rad Laboratories, Inc. (Hercules, CA, USA). Dexamethasone, ascorbic acid, β-glycerophosphate, Triton X-100, dimethyl sulfoxide and alizarin red S were purchased from Sigma (St. Louis, MO, USA). Hepcidin was purchased from Peptide Institute, Inc. (Osaka, Japan). Anti-β-actin (sc-7210), anti-IgG HRP (sc-2004), anti-phosphorylated (p)-extracellular signal-related kinase (ERK) (sc-292838), anti-p-P38 (101759) and anti-p-c-Jun N-terminal kinase (JNK) (sc-135642) were purchased from Santa Cruz Biotechnology, Inc. (Santa Cruz, CA, USA). Anti-bone morphogenetic protein (BMP)2 (ab14933), anti-alkaline phosphatase (ALP) (ab955462), anti-osteocalcin (OCN) (ab13418) and anti-p-small mothers against decapentaplegic (Smad) 1, 5, and 8 (ab46688, ab13724 and ab3848) were purchased from Abcam (Cambridge, UK). The enhanced chemiluminescence (ECL) kit was purchased from Pierce Biotechnology, Inc. (Rockford, IL, USA). The ALP assay kit was purchased from Abcam. The nitrocellulose membrane was purchased from Millipore (Billerica, MA, USA). All water used was glass distilled.

### Cell culture

Mesenchymal stem cells were collected from Sprague Dawley (SD) rats by flushing the femora with α-MEM supplemented with 10% FBS, 100 U/ml penicillin and 100 μg/ml streptomycin. The obtained suspension was centrifuged at 300 × g for 5 min. The pellet was resuspended in α-MEM supplemented with 10% FBS, 100 U/ml penicillin, and 100 μg/ml streptomycin and incubated in 5% CO_2_ at 37°C for 72 h. Since the MSCs were able to adhere to the surface of the culture dishes unlike hemopoietic cells, the adherent cells were isolated from the bone marrow through adherence-separation culturing. The primary cells were passaged after ~4 days (7 days from isolation), when the MSCs had reached 80% confluence. The adherent cells were retrieved by trypsinization and then replated.

### Osteogenic differentiation

To assess the osteogenic differentiation of the osteoblasts, the cells were seeded into 24-well plates or 10 cm culture dishes and, when the cells reached 80% confluence, they were treated with osteogenic medium consisting of phenol red-free α-MEM, 10% FBS, dexamethasone (1×10^−8^ M), ascorbic acid (50 μg/ml) and β-glycerophosphate (1×10^−2^ M). The medium was replaced at three day intervals.

### Alizarin red S staining

Deposition of intracellular calcium in the osteoblasts was assessed under different conditions using alizarin red S staining. Briefly, the MSCs were seeded into 24-well plates and grown under osteogenic conditions, as described above. On different culture days, the cells were fixed using 4% paraformaldehyde, washed with sterile 1× phosphate-buffered saline, stained using 2% (w/v) alizarin red S and visualized using light microscopy (ECLIPSE TE2000-U, Nikon, Tokyo, Japan).

### ALP detection

The MSCs cells were lysed by incubation in 1% Triton X-100 in DPBS on ice for 1.5 h. Protein solutions were centrifuged at 2,000 × g for 15 min at 4°C, and the activity of ALP in the lysate was detected using an ALP assay kit (Abcam) according to the manufacturer’s instructions. The absorbance of the lysates was measured by spectrophotometer (DU 640, Beckman Coulter, Miami, FL, USA) at a wavelength of 405 nm. The corresponding activity was calculated based on a standard curve according to a sample absorbance value.

### Reverse transcription quantitative polymerase chain reaction (RT-qPCR)

Total RNA from the cultured cells was isolated using TRIzol reagent (Invitrogen Life Technologies). RNase-free DNaseI was used to eliminate genomic DNA contamination in the RNA samples and the 260/280 absorbance ratio was measured by spectrophotometer (DU 640, Beckman, USA) to verify the purity of the RNA. Gene sequences for SMP2 and GAPDH were obtained from the GenBank database, and specific primers were designed for each over an exon-exon junction using Primer Premier 5.0 software (Premier Biosoft International, Palo Alto, CA, USA). The following primers were used: GAPDH, 5′-GATGCTGGTGCTGAGTATGRCG-3′, 5′-GTGGTGCAGGATGCATTGCTCTGA-3′; BMP2, 5′-CACGGATCCCAAGCCAAACACAAACAGCGGAA-3′, 5′-GTGAAGCTTCTAGCGACACCCACAACCCTCC-3′; Smad1, 5′-TTTCTGAAACTGTATGCTGGCTGTATTACT-3′, 5′-CCA GTAGAGAAAAACCCTGCTAGTGTTGG-3′; Smad5: 5′-GTGTATAAATCGGCATGAGTAGCTATCC-3′, 5′-GGCTTTGAGAGCACAATACAGTAAAACC-3′ and Smad8, 5′-ACACAGCGAGTACAACCCTCAGC-3′, 5′-GTTCGTAGTAGGCAACAGAACACCAGTG C-3′. The RT-qPCR reactions were performed using the Gene Amp PCR system 9700 (Perkin-Elmer, Foster City, CA, USA) and amplified for 35 cycles. RT-qPCR reactions were performed under the condition of 94°C for 4 min; 35 cycles of 94°C for 30 sec, 55°C for 30 sec, 72°C for 1 min; final extension 72°C for 10 min. The amplified products were then separated by electrophoresis on a 2% agarose gel and visualized using ethidium bromide staining (Bio-Rad Laboratories, Inc.). Each product was visualized following separation using GAPDH as an internal control. The image density was quantified using a FluoroImager SI (Amersham Pharmacia Biotech, Amersham, UK).

### RNA interference, plasmid construction and transient transfection

The control small interfering (si)RNA and the siRNA duplexes specific for BMP2 (siBMP2) were transfected into the MSCs using Lipofectamine 2000 (Invitrogen Life Technologies) according to the manufacturer’s instructions. siBMP2 (cat no. sc-39738) was purchased from Santa Cruz Biotechnology, Inc. All experiments were performed in 6-well tissue culture plates and tranfection was carried out when the plated cells had reached 50–60% confluence. The transfection efficiency averaged between 50–70%. The cells were left to recover in the medium for 24 h following transfection.

### Western blot analysis

The cells were lysed using a mixture of radioimmunoprecipitation assay buffer and phenylmethylsulfonyl fluoride (1:100) on ice for 30 min with occasional mixing. The lysed cells were sonicated by SONICS VC130PB (Sonics and Materials, Inc., Newtown, CT, USA). Cell extracts were prepared by repeated freeze/thaw 5 times and sonicated at 200W for 5 sec three times with a 30 sec incubation on ice between bursts and centrifuged at 10,800 × g at 4°C for 5 min. The total protein concentration was measured using the Bradford method (19). Samples containing 10 μg protein were electrophoresed and then transferred onto nitrocellulose membranes (Millipore). The nitrocellulose membrane was cut according to the molecular weight of the protein and was incubated with different protein antibodies for overnight at 4°C. The primary antibodies used in the present study were as follows: anti-ALP (1:400), anti-OCN (1:400), anti-BMP2 (1:400), anti-p-Smad1 (1:400), anti-p-Smad5 (1:400), anti-p-Smad8 (1:400), anti-p-ERK (1:400), anti-p-P38 (1:400) and anti-p-JNK (1:400). The appropriate peroxidase-conjugated secondary antibodies were used and incubated at room temperature for 1 h, and detection was performed using an ECL kit (Pierce Biotechnology, Inc.). The relative quantities of the various proteins were analyzed and the results were quantified using Quantity One software (Bio-Rad, Hercules, CA, USA).

### Statistical analysis

Statistical analysis was performed using SPSS version 18 (SPSS, Inc., Chicago, IL, USA). The data are expressed as the means ± standard error of the mean. The variance was homogenous and a standard analysis of variance (ANOVA) methodology was used. Following the establishment of statistical significance using ANOVA, individual comparisons were made using Tukey’s multiple comparison test. P<0.05 was considered to indicate a statistically significant difference.

## Results

### Effect of hepcidin on osteogenic differentiation in MSCs

**Figure 1:**
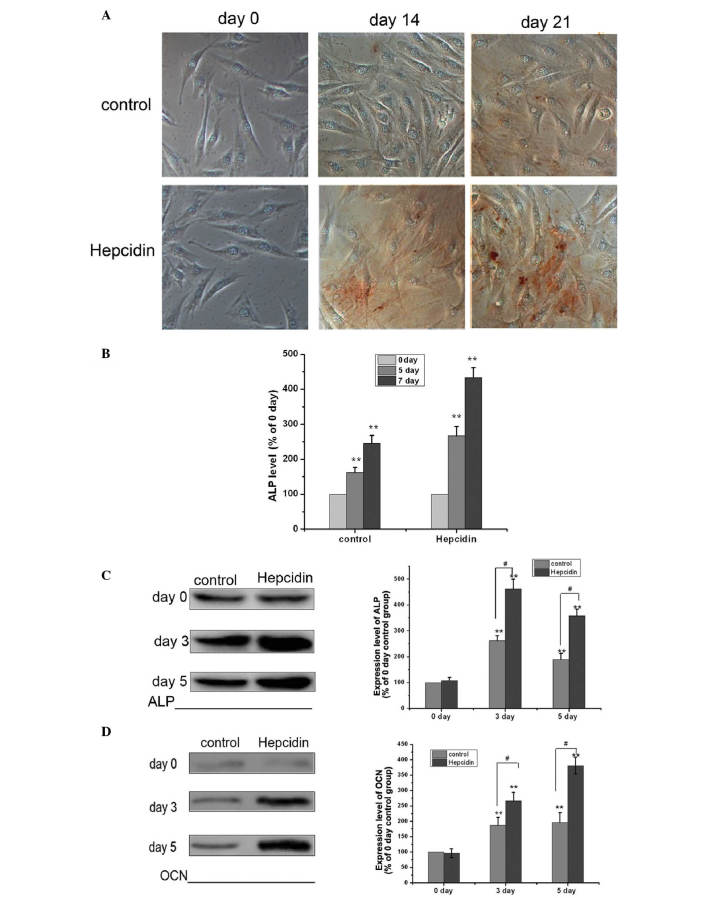
Effect of hepcidin on the osteogenic differentiation and protein expression of ALP and OCN and mineralization in MSCs. (A) MSCs were cultured in osteogenic medium with or without 0.2 mmol/l hepcidin for 0, 14 and 21 days to induce osteogenic differentiation. Intracellular calcium deposition was assessed using alizarin red staining (magnification, ×100). (B) MSCs were cultured in osteogenic medium with or without hepcidin for 0, 5 and 7 days and the levels of ALP were detected using an ALP assay (^**^P<0.01, vs.0 days). (C) MSCs were cultured in osteogenic medium with or without hepcidin for 0, 3 and 5 days and the levels of ALP were detected by western blotting (^**^P<0.01, vs. 0 days; ^#^P<0.01 hepcidin group, vs. control group). (D) MSCs were cultured in osteogenic medium with or without hepcidin for 0, 3 and 5 days and the levels of OCN were detected by western blotting (^**^P<0.01, vs. 0 days; ^#^P<0.01 hepcidin group, vs. control group). MSC, mesenchymal stem cell; ALP, alkaline phosphatase; OCN, osteocalcin.

To evaluate the effect of hepcidin on osteogenic differentiation, a critical function, MSCs were cultured in osteogenic medium in the presence or absence of 0.2 mmol/l hepcidin for 0, 14 and 21 days. The concentration of hepcidin used was that used in a previous study (20). The present study demonstrated that the intercellular accumulation of calcium was markedly increased compared with the control group (Fig. 1A). The osteogenic differentiation ability of MSCs from control and hepcidin groups was then evaluated and compared by detecting the activity of ALP and the expression of OCN. As shown in Fig. 1B and C, the ALP assay and western blot analysis revealed that the MSCs cultured with hepcidin exhibited a significantly higher ALP level compared with the control groups following culture for 3, 5 and 7 days. Similarly, the expression of OCN was greater in the hepcidin groups compared with the control groups. Taken together, these findings indicated that, under hepcidin culture conditions, the osteogenic differentiation of MSCs was significantly improved.

### Hepcidin activates the BMP2/Smad pathway in MSCs

**Figure 2:**
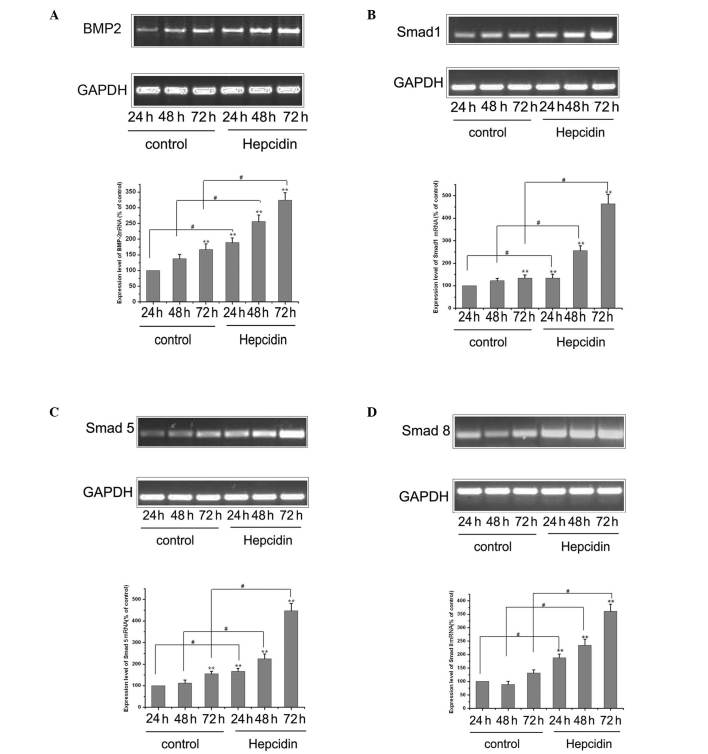
Effect of hepcidin on the BMP2/Smads pathway in MSCs. (A) MSCs were cultured in osteogenic medium with or without 0.2 mmol/l hepcidin for 24, 48 and 72 h and the expression level of BMP2 mRNA was detected using RT-qPCR. The data are expressed as the means ± SEM (n=3). GAPDH was used as a loading control (^**^P<0.01, vs. control; ^#^P<0.01, vs. control). (B) Cells were cultured, as described above, and the expression level of Smad1 mRNA was detected using RT-qPCR. The data are expressed as the means ± SEM (n=3). GAPDH was used as a loading control. (^**^P<0.01, vs. control; ^#^P<0.01, vs. control). (C) Cells were cultured, as described above, and the expression level of Smad5 mRNA was detected using RT-qPCR. The data are expressed as the means ± SEM (n=3). GAPDH was used as a loading control (^**^P<0.01, vs. control 24 h; ^#^P<0.01, vs. control). (D) Cells were cultured, as described above, and the expression level of Smad8 mRNA was detected using RT-qPCR. The data are expressed as the means ± SEM (n=3). GAPDH was used as a loading control (^**^P<0.01, vs. control; ^#^P<0.01, vs. control). MSC, mesenchymal stem cell; BMP2, bone morphogenetic protein 2; Smads; small mothers against decapentaplegic; RT-qPCR, reverse transcription polymerase chain reaction; SEM, standard error of the mean.

Previous studies have demonstrated that BMP/Smad signaling pathway usually mediates osteoblastic differentiation and maturation (21). Therefore, the present study examined whether the treatment of MSCs with hepcidin affected the BMP/Smad pathways in this process. The MSCs were cultured in osteogenic medium with or without 0.2 mmol/l hepcidin for 24, 48 and 72 hours, The expression levels of BMP2 mRNA, Smad1 mRNA, Smad5 mRNA and Smad8 mRNA were detected using RT-qPCR. As shown in Fig. 2, the BMP2, Smad1, Smad5 and Smad8 mRNAs were significantly upregulated by hepcidin treatment compared with the control groups, following culture for 24, 48 and 72 h. Overall, these results suggested that hepcidin activated the BMP2/Smad pathway.

### Hepcidin promotes osteogenic differentiation via the BMP2/Smad pathway in MSCs

**Figure 3:**
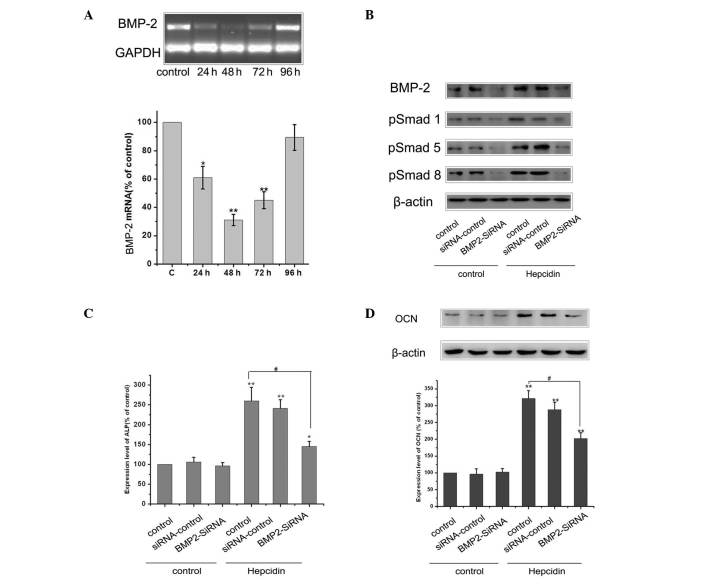
BMP2/Smad signaling pathways contribute to hepcidin-induced osteogenic differentiation by hepcidin. (A) RT-qPCR analysis was performed using total RNA isolated from BMP2 mRNA following BMP2-siRNA transfection at different times in the MSCs. Each value is expressed as the mean ± SEM (n=3). GAPDH was used as a loading control (^*^P<0.05, vs. control; ^**^P<0.01, vs. control). (B) Cells in the control, siRNA control and BMP2-siRNA groups were cultured in osteogenic medium with or without 0.2 mmol/l hepcidin for 48 h and the expression level of p-Smad1, p-Smad5 and p-Smad8 were detected by western blotting. Each value is expressed as the mean ± SEM (n=3). β-actin was used as a loading control. (C) Cells were cultured, and the expression levels of ALP were detected using an ALP assay. Each value is expressed as the mean ± SEM (n=3; ^*^P<0.05, vs. control; ^**^P<0.01, vs. control; ^#^P<0.01, vs. hepcidin + BMP2-siRNA group). (D) Cells were cultured, as described above, and the expression levels of OCN were detected by western blotting. Each value is expressed as the mean ± SEM (n=3). β-actin was used as a loading control. (^*^P<0.01, vs. control; ^#^P<0.01, vs. hepcidin + BMP2-siRNA group). MSC, mesenchymal stem cell; BMP2, bone morphogenetic protein 2; Smad; small mothers against decapentaplegic; p-Smad, phosphorylated Smad; RT-qPRC, reverse transcription polymerase chain reaction; SEM, standard error of the mean.

In order to examine whether hepcidin promoted osteogenic differentiation through the BMP2/Smad pathways, the MSC cells were transfected with a plasmid expressing either the siRNA-BMP2 gene or siRNA-control. RT-qPCR analysis was performed using the total RNA isolated to determine the level of BMP2 mRNA following BMP2-siRNA and siRNA-control transfection at different times (Fig. 3A). When the cells were transfected with BMP2-siRNA, the expression of BMP2 was significantly decreased and reached the lowest value at 48 h compared with the control. The cells in the control, siRNA control and BMP2-siRNA groups were then cultured in osteogenic medium with or without 0.2 mmol/l hepcidin for 48 h and the expression levels of BMP2, p-Smad1, p-Smad5 and p-Smad8 were detected by western blot analysis. As shown in Fig. 3B, siRNA-BMP2 transfection significantly downregulated the levels of BMP2, p-Smad1, p-Smad5 and p-Smad8 in the presence of hepcidin treatment.

As shown in Fig. 1, under hepcidin culture conditions, the osteogenic differentiation ability of the MSCs significantly improved. However, the activation effect of hepcidin was significantly reversed when the cells were transfected with BMP2 siRNA. As shown in Fig. 3C and D, hepcidin significantly upregulated the level of ALP and OCN compared with the control. By contrast, the siRNA-mediated suppression of BMP2 inhibited the osteogenic differentiation induced by hepcidin, resulting in a significant decrease in ALP and OCN. These data demonstrated that the promotion of osteogenic differentiation by hepcidin may be mediated by the BMP2/Smad pathway.

### Hepcidin promotes osteogenic differentiation by the mitogen-activated protein kinase (MAPK)/P38 pathway in MSCs

**Figure 4:**
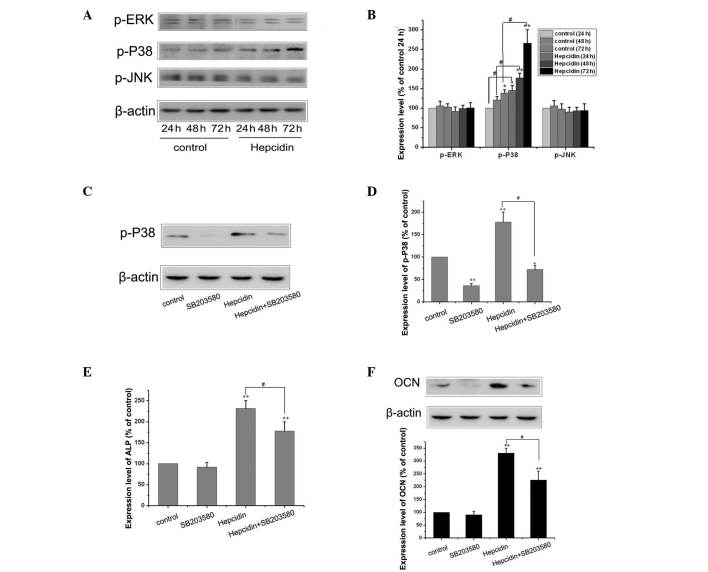
MAPK/P38 signaling pathways contribute to osteogenic differentiation induced by hepcidin. (A) Cells in the control, siRNA control group and BMP2-siRNA groups were cultured in osteogenic medium with or without 0.2 mmol/l hepcidin for 48 h and the expression levels of p-ERK, p-P38 and p-JNK were detected using wetern blot analysis. (B) Each value is expressed as the mean ± SEM (n=3) and β-actin was used as a loading control (^*^P<0.05, vs. control; ^**^P<0.01, vs. control; ^#^P<0.01, vs. hepcidin. (C) Cells were cultured in osteogenic medium and exposed to 0.2 mmol/l hepcidin with or without 10 μM SB203580 for 48 h and the expression levels of p-P38 were detected by western blotting. (D) Each value is expressed as the mean ± SEM (n=3) and β-actin was used as a loading control (^*^P<0.05, vs. control; ^**^P<0.01, vs. control; ^#^P<0.01 hepcidin, vs. hepcidin + SB203580). (E) Cells were cultured, as described above, and the expression levels of ALP were detected using an alkaline phosphatase assay kit. Each value is expressed as the mean ± SEM (n=3; ^**^P<0.01, vs. control; ^#^P<0.01 hepcidin, vs. hepcidin + SB203580). (F) Cells were cultured, as described above, and the expression levels of OCN were detected by western blotting. Each value is expressed as the mean ± SEM (n=3) and β-actin was used as a loading control (^**^P<0.01, vs. control; ^#^P<0.01 hepcidin, vs. hepcidin + SB203580). MSC, mesenchymal stem cell; MAPK, mitogen-activated protein kinase; RT-qPRC, reverse transcription polymerase chain reaction; SEM, standard error of the mean.

It has been previously demonstrated that the MAPK signaling pathway is involved in the osteogenic differentiation of MSCs (22). To investigate the possible role of the MAPK signaling pathway in the effect of hepcidin, the MSCs were cultured in osteogenic medium with or without 0.2 mmol/l hepcidin for 24, 48 and 72 h and the expression levels of p-ERK, p-P38 and p-JNK were analyzed by western blotting. As shown in Fig. 4A and B, the level of p-P38 was significantly upregulated by hepcidin compared with the control group following culture for 24, 48 and 72 h. However, no significant change was observed in the levels of p-ERK and p-JNK.

The present study hypothesized that the MAPK/P38 signaling pathway may be important in the process of osteogenic differentiation of MSCs. Therefore, 10 μM SB203580, an inhibitor of the MAPK/P38 pathway (23), was added to the culture medium of the cells. As shown in Fig. 4C and D, hepcidin significantly upregulated the level of p-P38, However, the activation effect of hepcidin was significantly reversed following pre-treatment of the cells with SB203580. Similarly, inhibition of the P38 pathway by SB203580 also inhibited the promotion of osteoblast differentiation by hepcidin (Fig. 4E and F). These data demonstrated that the promotion of osteogenic differentiation by hepcidin may be mediated by the MAPK/P38 pathway.

## Discussion

The investigation of osteogenic differentiation to identify biologically active anti-osteoporotic agents is a complicated process and is governed by numerous regulators. Although its precise mechanism remains to be elucidated, this biological processes is believed to be important in bone differentiation and mineralization. Substantial investigation has been performed to gain an improved understanding of the process of bone formation and to identify methods to promote osteogenic differentiation. MSCs are multipotent stromal cells, which have self-renewal capacity and are able to differentiate into a variety of mesenchymal lineages, including adipocytes, osteoblasts and chondrocytes (24). In the present study, osteogenic medium containing dexamethasone, ascorbic acid and β-glycerophosphate, was used to induce osteogenic differentiation in MSC cells.

Hepcidin is a peptide hormone, which is secreted mainly by hepatocytes in the liver and the expression of which is positively regulated by iron-loading in the body (25,26). Hepcidin increases intracellular iron by binding to and degrading ferroportin and increases intracellular calcium, which directly promotes the deposition of calcium and indirectly contributes to the function of osteoblasts (27). However, the effect of hepcidin on osteogenic differentiation in MSCs remains to be elucidated. In the present study, it was demonstrated that hepcidin induced osteoblast differentiation in the MSCs. As shown in Fig. 1, the MSCs cultured with hepcidin exhibited significantly higher levels of ALP and OCN as compared with the control groups. The BMP2-dependent Smad and MAPK/P38 signaling pathways are two major pathways involved in hepcidin-induced osteogenic transcriptional networks (28,29). BMP2 is a member of the transforming growth factor-β superfamily, which are key regulators involved in bone formation and are important in the differentiation process of MSCs to osteoblast-like cells (30). Hepcidin is closely associated with BMP2 and a previous study demonstrated that the BMP2/4-dependent pathway regulates the expression of hepcidin and that BMP2/4 are components of the iron-sensing and signaling pathway, which regulates hepcidin synthesis (31,32). The present study aimed to identify whether hepcidin had feedback effects on the BMP2/Smad pathways, since BMP2/Smad lead to positive effects on bone formation. The present study demonstrated that hepcidin had positive effect on the expression of BMP2, which was confirmed using western blot analysis to assess the level of BMP2 and Smad1,5,8. The results suggested that hepcidin induced the differentiation of the MC3T3-E1 osteoblastic cells via the BMP2/Smad pathway, as the siRNA-mediated suppression of BMP2 inhibited the effect of hepcidin.

P38 MAPKs are members of the MAPK family and are involved in osteoblast differentiation (33). To investigate the involvement of MAPKs in hepcidin-induced osteoblast differentiation, the expression levels of p-ERK, p-P38 and p-JNK were detected. No significant change was observed in the ERK or JNK pathways, however upregulation of p-P38 was observed following culture with hepcidin. Therefore, the present study investigated whether the MAPK/p38 pathway was associated with hepcidin-induced osteoblast differentiation. Western blot analyses revealed that hepcidin was able to phosphorylate p38 in the MSC cells (Fig. 4). In addition, the hepcidin-induced differentiation and mineralization of MSC cells was reversed in the presence of SB203580, a specific MAPK/p38 inhibitor. These results suggested that MAPK/p38 signaling pathway was responsible for hepcidin-induced osteoblastic differentiation and osteogenic gene expression (Fig. 4).

In conclusion, hepcidin was observed to induce the osteogenic differentiation of MSC cells via activation of the BMP2/Smad and MAPK/p38 pathways. Hepcidin may, therefore, possess therapeutic potential for treatment of osteoporosis by promoting bone formation.

## Abbreviations

MSCs: mesenchymal stem cells
BMP: bone morphogenetic proteins
SMADs: small mothers against decapentaplegic homologs
α-MEM: α-minimal essential medium
FBS: fetal bovine serum
MAPK: mitogen-activated protein kinase
ERK: extracellular signal-regulated-kinase
JNK: c-jun N-terminal kinase
